# Author Correction: A model for the fragmentation kinetics of crumpled thin sheets

**DOI:** 10.1038/s41467-021-22791-z

**Published:** 2021-04-15

**Authors:** Jovana Andrejevic, Lisa M. Lee, Shmuel M. Rubinstein, Chris H. Rycroft

**Affiliations:** 1 John A. Paulson School of Engineering and Applied Sciences, Harvard University, Cambridge, MA USA; 2 The Racah Institute of Physics, The Hebrew University of Jerusalem, Jerusalem, 91904 Israel; 3 Computational Research Division, Lawrence Berkeley Laboratory, Berkeley, CA USA

**Keywords:** Applied mathematics, Scientific data, Statistical physics, thermodynamics and nonlinear dynamics

Correction to: *Nature Communications* 10.1038/s41467-021-21625-2, published online 5 March 2021.

The original version of this Article omitted DMR-1420570 from the Acknowledgements.

This has now been corrected in both the PDF and HTML versions of the Article.

