# Supplementary material for: A model for the fragmentation kinetics of crumpled thin sheets
Source: Nat Commun. 2021 Mar 5;12:1470. doi: 10.1038/s41467-021-21625-2 (PMC7935925; doi:10.1038/s41467-021-21625-2)
Supplement: Supplementary file 1 — Supplementary Information [file 41467_2021_21625_MOESM1_ESM.pdf]

A model for the fragmentation kinetics of crumpled thin sheets

Jovana Andrejevic,[1] Lisa M. Lee,[1] Shmuel M. Rubinstein,[2] and Chris H. Rycroft[1,3]
[1] *John A. Paulson School of Engineering and Applied Sciences, Harvard University, Cambridge, MA 02138, USA*
[2] *The Racah Institute of Physics, The Hebrew University of Jerusalem, Jerusalem 91904, Israel*
[3] *Computational Research Division, Lawrence Berkeley Laboratory, Berkeley, CA 94720, USA*

SUPPLEMENTARY FIGURE 1

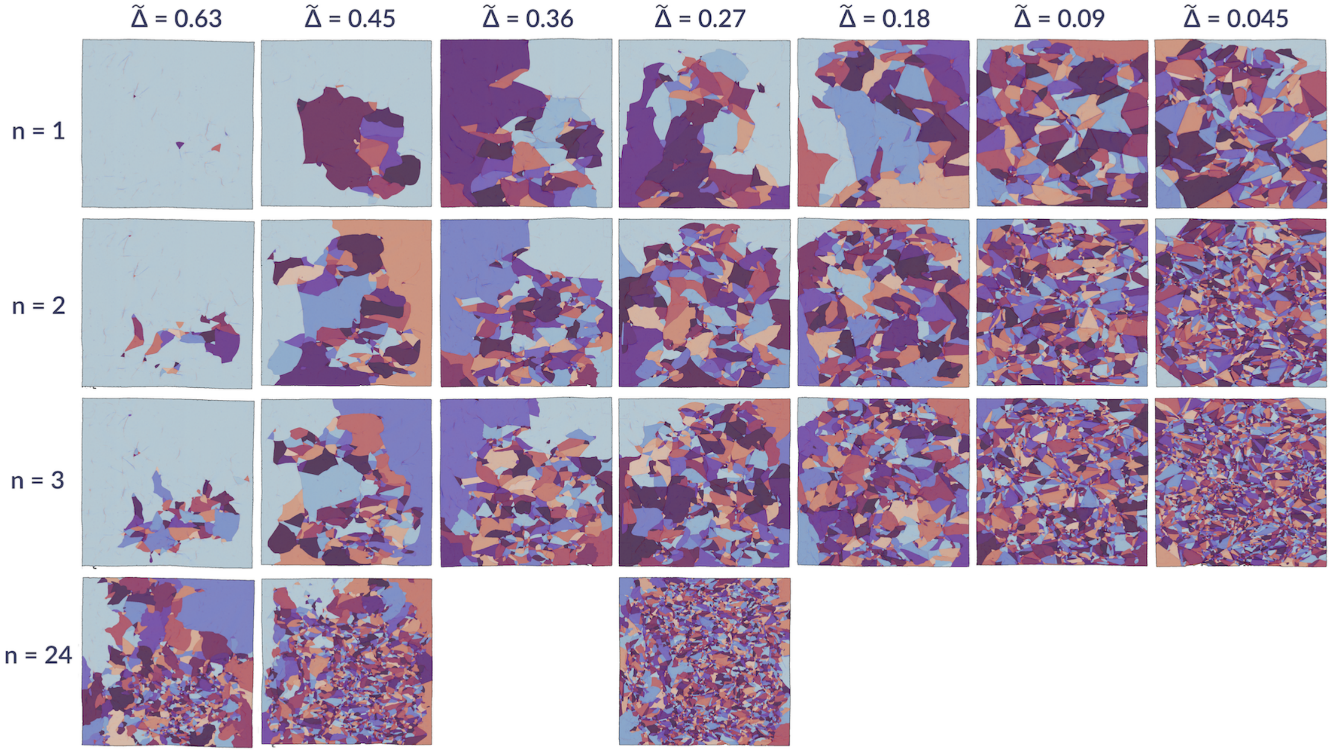

Supplementary Figure 1. **Manual facet segmentation.** Scans of crumpled sheets manually segmented into individual facets delineated by creases. Each column features a single sheet crumpled repeatedly n times to a specified compaction ratio $\tilde{\Delta} = L/L_0$. Random coloring is used for visual distinction between facets.

SUPPLEMENTARY FIGURE 2

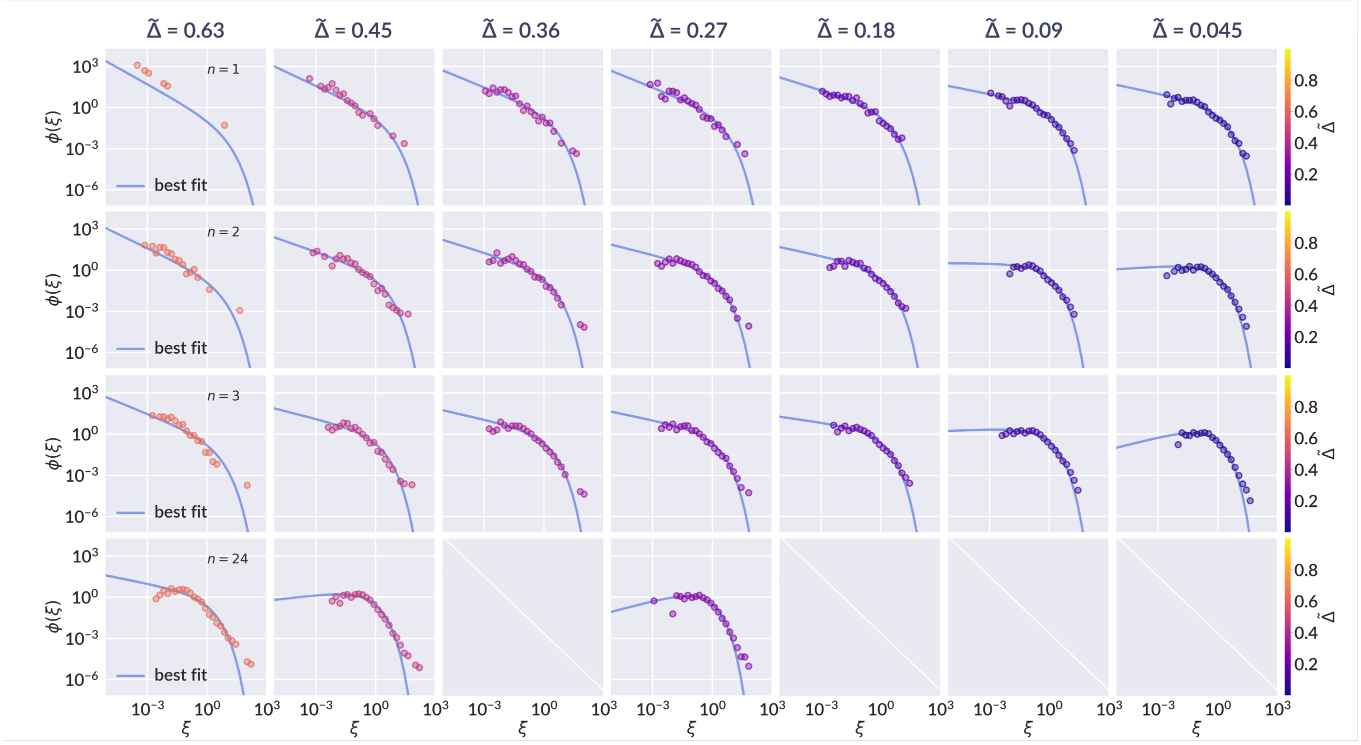

Supplementary Figure 2. **Facet area distributions from manual segmentation.** Distributions of facet areas x normalized by the mean area s respectively for each sample; $\xi = x/s$. The solid line shows the best fit to Supplementary Equation (3b), where the parameter a is calculated from the relation $a(t) = \sqrt{t/\tau}$ presented in the main text with single fitting parameter τ across the entire dataset. Marker colors correspond to different values of $\tilde{\Delta}$, as indicated by the colorbar.

SUPPLEMENTARY FIGURE 3

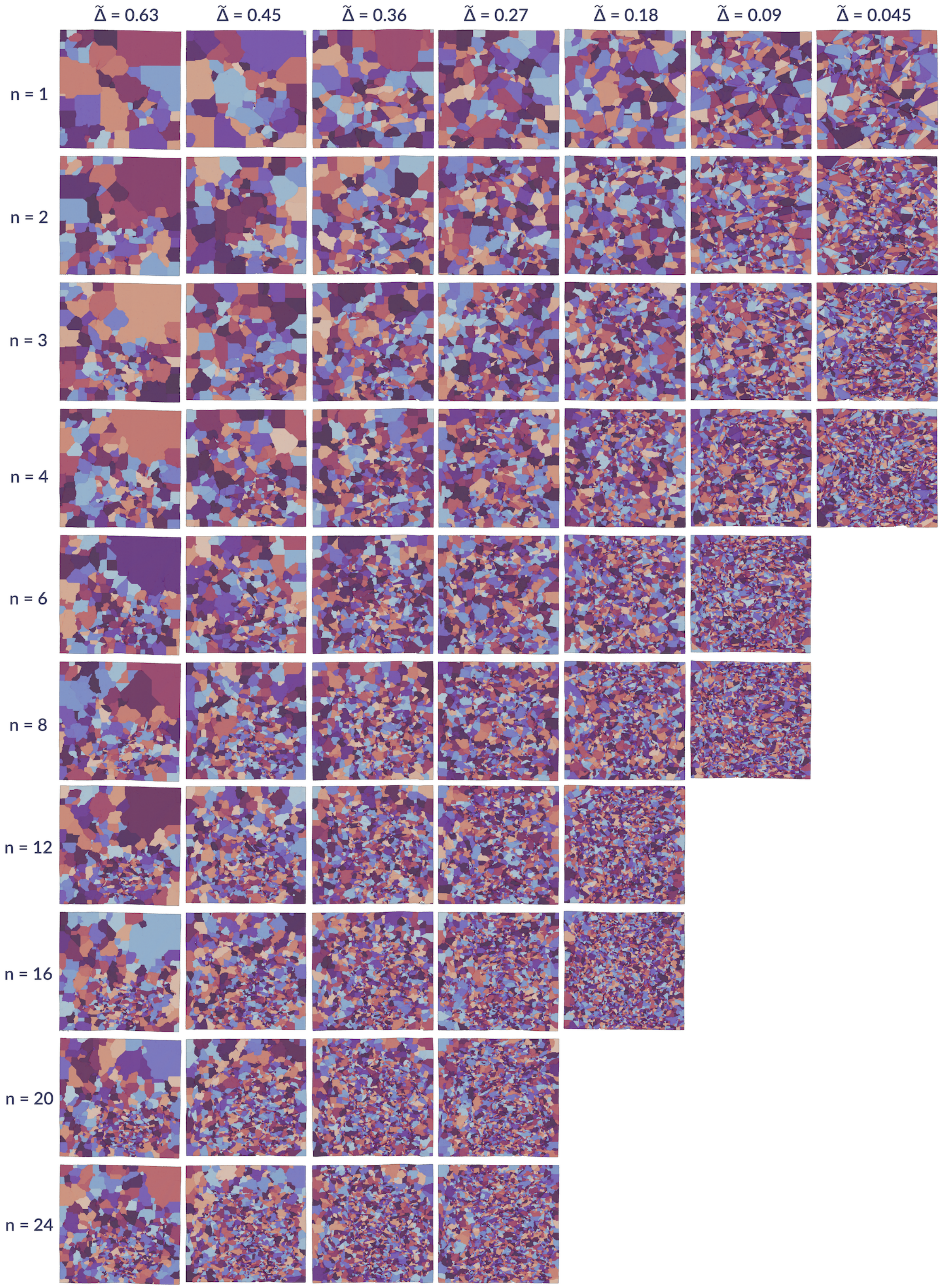

Supplementary Figure 3. **Automated facet segmentation.** Results of automated segmentation, shown for the same experimental samples as Supplementary Fig. 1, and including a larger representation of crumpling iterations. Random coloring is used for visual distinction between facets.

SUPPLEMENTARY FIGURE 4

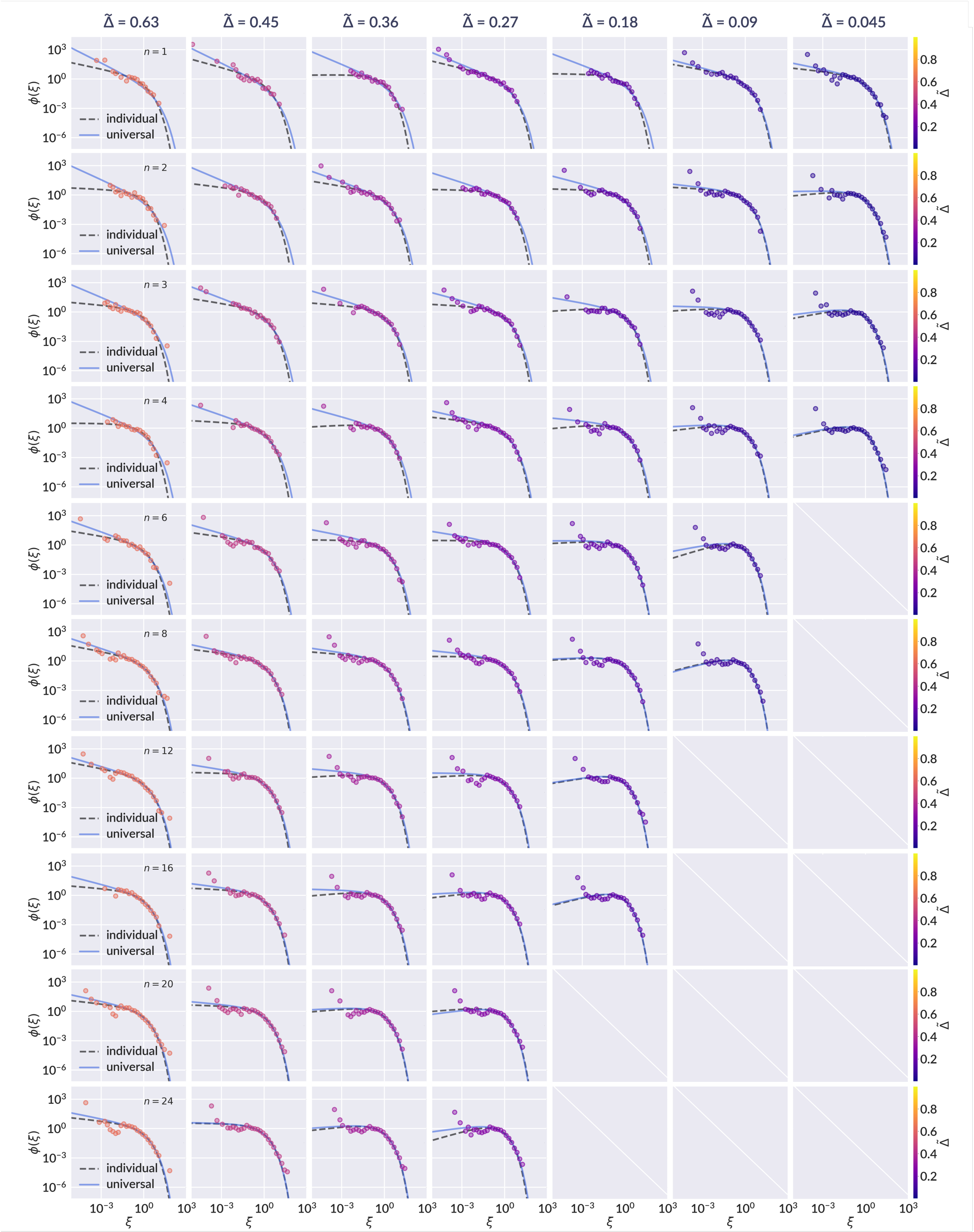

Supplementary Figure 4. **Facet area distributions from automated segmentation.** Corresponding distributions of normalized facet areas ξ for the data in Supplementary Fig. 3. The two accompanying curves show the best individual fit to Supplementary Equation (3b) with fitting parameter a (dashed line), and the curve obtained via the relation $a = \sqrt{t/\tau}$ with universal parameter τ fit from the manually segmented data (solid line). Marker colors correspond to different values of $\tilde{\Delta}$, as indicated by the colorbar. Due to the automated method's sensitivity to artifacts in crease detection, we see that weakly crumpled sheets tend to be over-partitioned, and the best fit a deviates from the predicted trend with t. However, denser crease networks demonstrate improved agreement between the individually fitted and predicted values of a.

SUPPLEMENTARY FIGURE 5

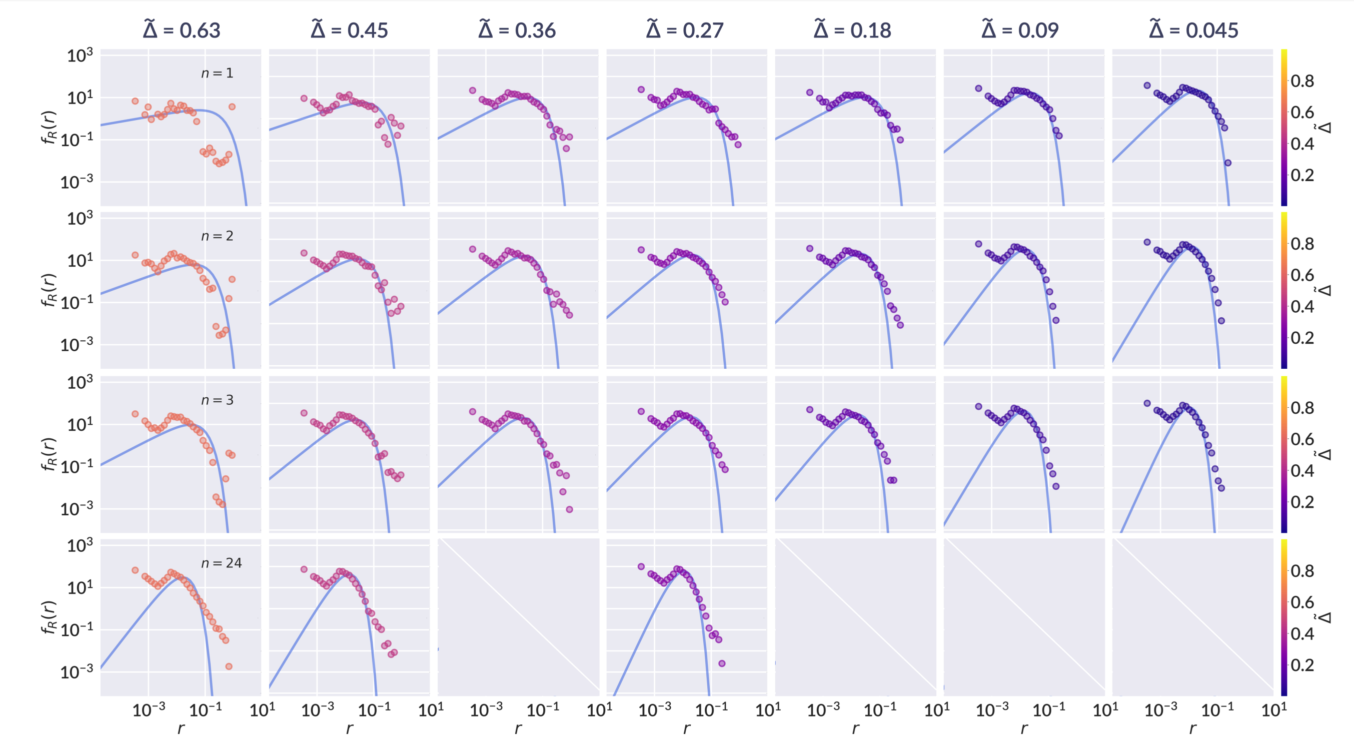

Supplementary Figure 5. **Facet length distributions from manual segmentation.** The distribution of facet lengths obtained by considering all vertical cross-sections of the facet patterns in Supplementary Fig. 1 (filled points), with the analytically derived distribution of Equation (15) of the main text plotted as a solid curve. Marker colors correspond to different values of $\tilde{\Delta}$, as indicated by the colorbar. Note that no additional fit is performed; the shape parameter a which appears in Equation (15) of the main text is the same as that obtained for the facet area distributions with a single fitting parameter τ, as detailed in the main text.

SUPPLEMENTARY FIGURE 6

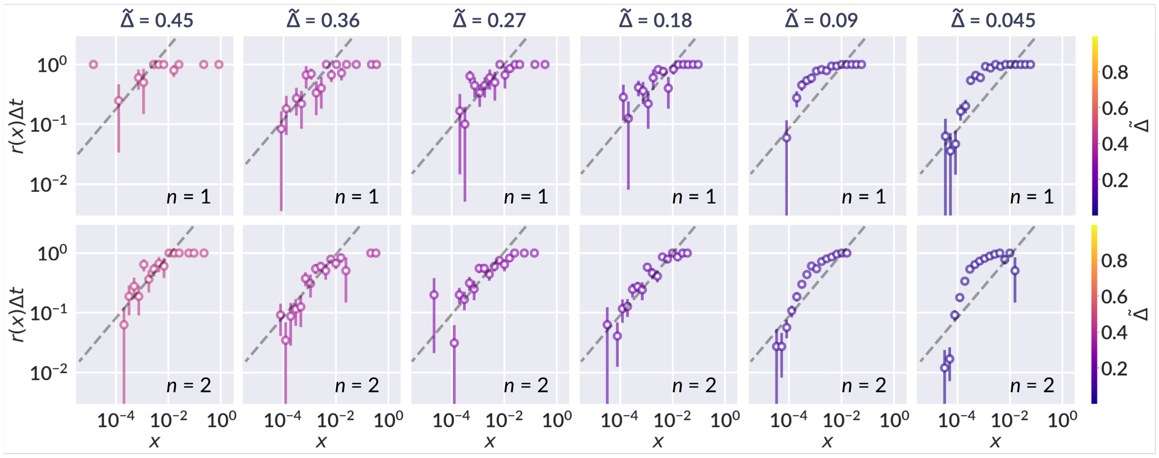

Supplementary Figure 6. **Estimation of overall breakup rate r(x).** For $n = 1$ (top row), the fraction of facets present at crumpling iteration $n = 1$ which fragment by $n = 2$, as a function of initial area x, for samples with $\tilde{\Delta} = 0.45, 0.36, 0.27, 0.18, 0.09,$ and 0.045 (across). For $n = 2$ (bottom row), the fraction of all facets present at crumpling iteration $n = 2$ which fragment by $n = 3$ for the same samples. The sample with $\tilde{\Delta} = 0.63$ had too few facets to form a sufficient representative sample. Error bars denote the standard deviation of the fragmentation probability if the fragmentation of each facet is regarded as a Bernoulli trial, with the fraction of fragmented facets taken as the success probability within each histogram bin. The dashed line corresponds to $\sqrt{x}$. Marker colors correspond to different values of $\tilde{\Delta}$, as indicated by the colorbar. As noted in the text, samples at small values of $\tilde{\Delta}$, or high compaction, likely undergo a succession of fragmentation events between $n = i$ and $n = i + 1$, and are thus poorer indicators of the statistics of single breakup events. Samples at large values of $\tilde{\Delta}$ are more likely resolve single breakup events, but have a lower population of facets from which to build the distribution. The choice of overall breakup rate $r(x) = x^{1/2}$ was motivated both by the stronger power law behavior at high $\tilde{\Delta}$, as well as its tractability in our analytical model.

SUPPLEMENTARY FIGURE 7

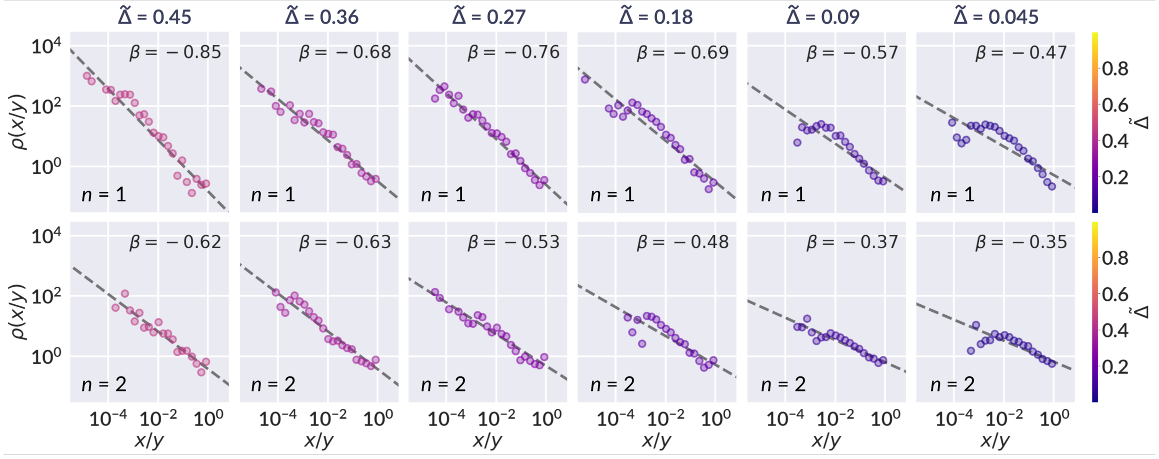

Supplementary Figure 7. **Estimation of conditional breakup probability f(x|y).** For $n = 1$ (top row), the probability density function $\rho(x/y)$ of facet areas x present in crumpling iteration $n = 2$ normalized by their parent facet's area y from $n = 1$, for samples with $\tilde{\Delta} = 0.45, 0.36, 0.27, 0.18, 0.09,$ and 0.045 (across). For $n = 2$ (bottom row), $\rho(x/y)$ of facet areas in $n = 3$ normalized by their parent facet's area from $n = 2$. Marker colors correspond to different values of $\tilde{\Delta}$, as indicated by the colorbar. The sample with $\tilde{\Delta} = 0.63$ had too few facets to form a sufficient representative sample and is excluded here. As noted in Supplementary Fig. 6, samples at small values of $\tilde{\Delta}$ likely undergo a succession of fragmentation events between crumples, and thus their distributions resemble the more mature facet distributions observed at later n, as in Supplementary Fig. 2.

SUPPLEMENTARY FIGURE 8

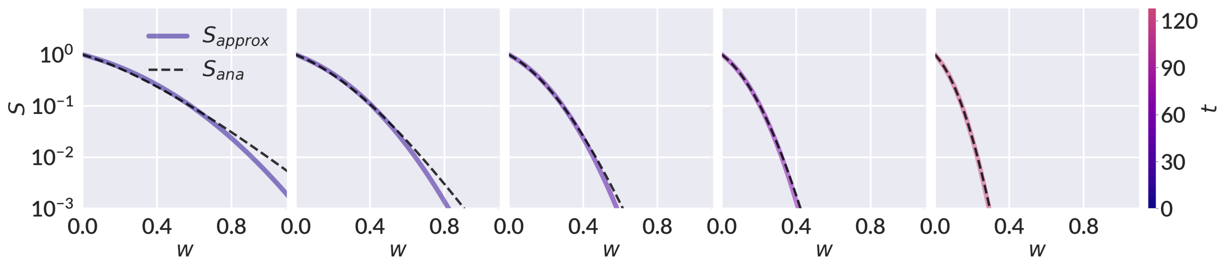

Supplementary Figure 8. **Convergence of asymptotic approximation for survival function $\mathbf{S_Z(w;\theta)}$.** Plot of Supplementary Equation (5) (dashed line) against the asymptotic approximation of Supplementary Equation (7) valid at large number of steps k (solid line), at $2k = 4, 8, 16, 32,$ and 64 steps from left to right, respectively. The shape parameter $a = 1$ for all cases, and t is appropriately determined from the relation $t = 2k(a+1)/L_0$. Curves of the asymptotic approximation are colored by the value of t, as indicated by the colorbar. The approximation shows increasing agreement with the exact solution for larger k, as anticipated.

SUPPLEMENTARY FIGURE 9

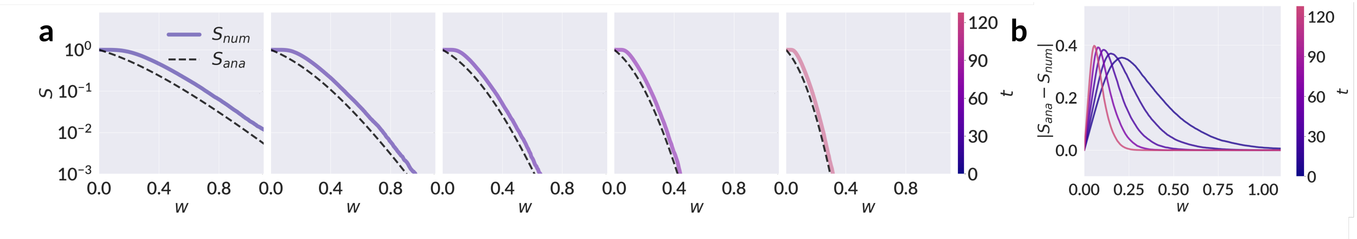

Supplementary Figure 9. **Numerical simulation of random walks and exact escape probability compared to analytical approximation. a** Plot of Supplementary Equation (5), which presents an analytical estimate of the fraction of escaped random walks looking only at final displacements (dashed line), compared against the more accurate result obtained by assessing all intermediate displacements through numerical simulation (solid line). For the latter, simulations of 50,000 random walks, with gamma-distributed steps sampled according to Supplementary Equation (4) with shape parameter $a = 1$, are performed with $2k = 4, 8, 16, 32,$ and 64 steps, and t appropriately determined from the relation $t = 2k(a+1)/L_0$. Curves corresponding to numerical simulation are colored by the value of t, as indicated by the colorbar. The analytical approximation systematically underestimates the number of escaped walks by approximately a constant factor; thus, $S_Z(w; a, \theta)$ remains proportional to the change in t over a crumpling iteration in both the analytically approximate and numerically calculated forms, allowing variation in the constant of proportionality. **b** The error between each pair of curves presented in **a**.

SUPPLEMENTARY METHODS

Manual segmentation. As noted in the main text, the final segmentation of all collected crease networks was performed by hand. These segmentations and the distributions of scaled facet areas are provided in Supplementary Figs. 1 & 2, respectively. We recognize in the Methods section of the main text that creases can soften over repeated crumples, and the result of unfolding and scanning between crumples could possibly contribute to the appearance of healing. To ensure that the crease patterns studied suitably fit the framework of a fragmentation model, we perform a simple analysis to affirm that healing is indeed very minimal. We make a quantitative prediction about the extent of healing by overlaying two manually segmented crease patterns from successive crumples $n-1$ and n of the same sheet, and measuring the length of creases present in $n-1$ which do not appear in n. We observe that the percent of healed creases make up less than 5% of any crease pattern; moreover, the fraction is typically within 1% for moderately to highly dense patterns. Thus, we conclude that healing is a small effect which does not greatly impact the dominant trends in the data.

Watershed segmentation. Prior to manual segmentation, an automated method using the watershed algorithm was initially tested. To perform this method, the maps of mean curvature for each sheet were first thresholded to produce a binary image separating creases, a pixel value of 1, and background, a pixel value of 0. The distance of each background pixel to its nearest crease pixel was then computed. The negative distance, which can be regarded as a topographic surface of hills and basins, was used as the elevation map for the watershed algorithm. The basins correspond to local minima of the surface, and regions centered at each basin are flooded until all pixels are assigned a basin. We identified pixels belonging to the same basin of the elevation map as a single facet of the crease pattern. However, several concerns prompted a more careful labeling by hand. Firstly, the separation of creases from background was performed using a custom technique referred to as the Radon transform method, detailed in the Supplementary Discussion of Ref. [1]. This technique combines global and local thresholding to accommodate variations in the intensity (curvature) of creases; nevertheless, softening of old creases near strongly imprinted ones weakens their detection. The watershed algorithm proved sensitive to creases which scar the sheet but do not form closed contours, particularly true at low confinement (high $\tilde{\Delta}$). Thus, the algorithm over-partitions the crease network in these cases. Mitigating the effect of smaller, isolated creases and vertices by stricter thresholding also compromises the detection of smaller facets, impacting densely scarred samples at low $\tilde{\Delta}$. The results of watershed segmentation and corresponding scaled facet area distributions are presented in Supplementary Figs. 3 & 4; while there is consistency with the manually segmented data, lower resolution and weaker performance for small features impacts the range over which consistent scaling is observed. Nevertheless, the automated method allows us to more easily segment a larger number of crease patterns; thus, we process a more extensive set of crumpling iterations for each compaction ratio considered.

SUPPLEMENTARY NOTE 1

Scaling solution to the fragmentation rate equation. Facet fragmentation is modeled following the theory of fragmentation kinetics outlined in Supplementary Ref. [2]. Here we reproduce the derivation of a scaling solution shared among setups with similar families of breakup rates, as well as carry out the analytical steps unique to our specific choice of these rates. The linear integro-differential equation describing the evolution of concentration of facet areas x, $c(x,t)$, is given by:

$$\frac{\partial c(x,t)}{\partial t} = \underbrace{-r(x)c(x,t)}_{\text{depletion of facets of area } x} + \int_x^\infty \underbrace{c(y,t)r(y)f(x|y)dy}_{\text{gain in facets of area } x}, \tag{1}$$

where

t	measure of progression of fragmentation
$r(x)$	overall rate at which a facet of area x breaks
$f(x\|y)$	conditional probability that x is produced from the breakup of y, $y \geq x$
$c(x,t)$	concentration of facets of area x

with the scaling ansatz

$$c(x,t) = \frac{1}{s^2}\phi\left(\frac{x}{s}\right),$$

which restricts all time dependence to a parameter $s = s(t)$ that represents the typical (mean) area, and $\phi(\xi)$ is a scaling function. The scaling function satisfies

$$\int_0^\infty \phi(\xi)d\xi = 1$$
$$\int_0^\infty \xi\phi(\xi)d\xi = 1$$

such that

$$\int_0^\infty c(x,t)dx = \frac{1}{s(t)} \qquad \text{(average number of facets)}$$
$$\int_0^\infty xc(x,t)dx = 1 \qquad \text{(total area)},$$

which ensures conservation of area. A common choice of $r(x)$ and $f(x|y)$ which prove analytically tractable are members of homogeneous kernels:

$$r(x) = x^\lambda,$$
$$f(x|y) = \frac{1}{y}b\left(\frac{x}{y}\right).$$

With this formulation, larger facets are more likely to split due to the higher rate given by $r(x)$ assuming $\lambda > 0$. Moreover, the conditional probability must satisfy area conservation,

$$\int_0^y xf(x|y)dx = y.$$

Plugging the scaling ansatz and general homogeneous kernels into the rate equation, and defining $\xi = x/s, \eta = y/s$ yields

$$\frac{\partial\left(\frac{1}{s^2}\phi(\xi)\right)}{\partial t} = -x^\lambda \frac{1}{s^2}\phi(\xi) + \int_x^\infty \frac{1}{s^2}\phi(\eta)y^\lambda \frac{1}{y}b\left(\frac{\xi}{\eta}\right)dy,$$
$$-\frac{2}{s^3}\dot{s}\phi(\xi) + \frac{1}{s^2}\phi'(\xi)\left(-\frac{x}{s^2}\right)\dot{s} = -s^{\lambda-2}\xi^\lambda\phi(\xi) + \int_\xi^\infty s^{\lambda-2}\phi(\eta)\eta^{\lambda-1}b\left(\frac{\xi}{\eta}\right)d\eta,$$
$$-\dot{s}s^{-(\lambda+1)}\Big(2\phi(\xi) + \xi\phi'(\xi)\Big) = -\xi^\lambda\phi(\xi) + \int_\xi^\infty \phi(\eta)\eta^{\lambda-1}b\left(\frac{\xi}{\eta}\right)d\eta,$$

where $\dot{s} \equiv ds/dt$. By separating the dependence on x and t we must have that

$$-\dot{s}s^{-(\lambda+1)} = \frac{-\xi^{\lambda}\phi(\xi) + \int_{\xi}^{\infty}\phi(\eta)\eta^{\lambda-1}b\left(\frac{\xi}{\eta}\right)d\eta}{2\phi(\xi) + \xi\phi'(\xi)} = \omega = \text{constant}$$

and thus have two equations

$$\omega\Big(2\phi(\xi) + \xi\phi'(\xi)\Big) = -\xi^{\lambda}\phi(\xi) + \int_{\xi}^{\infty}\phi(\eta)\eta^{\lambda-1}b\left(\frac{\xi}{\eta}\right)d\eta \tag{2a}$$

$$\dot{s}s^{-(\lambda+1)} = -\omega, \tag{2b}$$

Insight from experimental facet fragmentation data reveals a suitable form for the conditional breakup rate:

$$b\left(\frac{x}{y}\right) = \left(\frac{\beta+2}{\beta+1}\right)\rho\left(\frac{x}{y}\right),$$

where

$$\rho\left(\frac{x}{y}\right) = (\beta+1)\left(\frac{x}{y}\right)^{\beta}$$

with β a free parameter and $\rho(x/y)$ the probability density function of facet areas x normalized by their parent facet's area y from the previous crumpling iteration. In other words, $\rho(x/y)d(x/y)$ is the probability that a facet breaks to produce a fragment that is x/y of its initial area. This formulation introduces the assumption that fragmentation is a scale invariant process.

Next we demonstrate the agreement of our scaling function with the rate equation. We begin by re-expressing β in terms of a new parameter a as

$$\beta = \frac{a}{2} - 1.$$

Our proposed solution $\phi(\xi)$ takes the form

$$\phi(\xi) = \frac{\lambda}{\Gamma\left(\frac{a}{2\lambda}\right)}G(a,\lambda)\big(G(a,\lambda)\xi\big)^{\frac{a}{2}-1}e^{-\left(G(a,\lambda)\xi\right)^{\lambda}},$$

and thus

$$\begin{aligned}
\phi'(\xi) &= \frac{\lambda}{\Gamma\left(\frac{a}{2\lambda}\right)}G^2(a,\lambda)\left[\left(\frac{a}{2}-1\right)\big(G(a,\lambda)\xi\big)^{\frac{a}{2}-2} - \lambda\big(G(a,\lambda)\xi\big)^{\frac{a}{2}+\lambda-2}\right]e^{-\left(G(a,\lambda)\xi\right)^{\lambda}} \\
&= \left[\left(\frac{a}{2}-1\right) - \lambda\big(G(a,\lambda)\xi\big)^{\lambda}\right]\frac{\phi(\xi)}{\xi}
\end{aligned}$$

for $G(a,\lambda) = \Gamma\left(\frac{a+2}{2\lambda}\right)/\Gamma\left(\frac{a}{2\lambda}\right)$. Substituting in $b(x/y)$, we have that

$$\begin{aligned}
\int_{\xi}^{\infty}\phi(\eta)\eta^{\lambda-1}b\left(\frac{\xi}{\eta}\right)d\eta &= \frac{\lambda}{\Gamma\left(\frac{a}{2\lambda}\right)}G(a,\lambda)\left(\frac{a}{2}+1\right)\big(G(a,\lambda)\xi\big)^{\frac{a}{2}-1}\int_{\xi}^{\infty}\eta^{\lambda-1}e^{-\left(G(a,\lambda)\eta\right)^{\lambda}}d\eta \\
&= \frac{1}{\Gamma\left(\frac{a}{2\lambda}\right)}\Big(G(a,\lambda)\Big)^{1-\lambda}\left(\frac{a}{2}+1\right)\big(G(a,\lambda)\xi\big)^{\frac{a}{2}-1}e^{-\left(G(a,\lambda)\xi\right)^{\lambda}} \\
&= \frac{1}{\lambda}\Big(G(a,\lambda)\Big)^{-\lambda}\left(\frac{a}{2}+1\right)\phi(\xi)
\end{aligned}$$

Supplementary Equation (2a) may be solved to obtain

$$\begin{aligned}
\omega\left(2\phi(\xi) + \left[\left(\frac{a}{2}-1\right) - \lambda\big(G(a,\lambda)\xi\big)^{\lambda}\right]\phi(\xi)\right) &= -\xi^{\lambda}\phi(\xi) + \frac{1}{\lambda}\Big(G(a,\lambda)\Big)^{-\lambda}\left(\frac{a}{2}+1\right)\phi(\xi), \\
\omega\left(\frac{a}{2}+1-\lambda\big(G(a,\lambda)\xi\big)^{\lambda}\right)\phi(\xi) &= \frac{1}{\lambda}\Big(G(a,\lambda)\Big)^{-\lambda}\left(\frac{a}{2}+1-\lambda\big(G(a,\lambda)\xi\big)^{\lambda}\right)\phi(\xi)
\end{aligned}$$

which is solved for all ξ when

$$\omega = \frac{1}{\lambda}\Big(G(a,\lambda)\Big)^{-\lambda}.$$

Moving to Supplementary Equation (2b), we therefore have that

$$\begin{aligned}
\dot{s}s^{-(\lambda+1)} &= -\frac{1}{\lambda}\Big(G(a,\lambda)\Big)^{-\lambda}, \\
\int_{s_0}^{s} s'^{-(\lambda+1)}ds' &= -\int_0^t \frac{1}{\lambda}\Big(G(a,\lambda)\Big)^{-\lambda}dt', \\
-\frac{1}{\lambda}\Big(s^{-\lambda} - s_0^{-\lambda}\Big) &= -\frac{1}{\lambda}\Big(G(a,\lambda)\Big)^{-\lambda}t, \\
s &= \left(\Big(G(a,\lambda)\Big)^{-\lambda}t + s_0^{-\lambda}\right)^{-1/\lambda}
\end{aligned}$$

noting that $\lambda > 0$ in the case considered. The initial condition $s_0 = 1$ may be substituted for a fragmentation process originating from a single facet. However, in the limit of large t, the dependence of the typical area becomes insensitive to the initial condition, and our result may be simplified to $s(t) = \Big(G(a,\lambda)\Big)t^{-1/\lambda}$.

For the special case of $\lambda = 1/2$ considered in the main results of this work,

$$G\left(a, \lambda = \frac{1}{2}\right) = a(a+1), \tag{3a}$$

$$\phi(\xi) = \frac{a(a+1)}{2\Gamma(a)}\big(a(a+1)\xi\big)^{\frac{a}{2}-1}e^{-\sqrt{a(a+1)\xi}}, \tag{3b}$$

$$s(t) = \frac{a(a+1)}{t^2}. \tag{3c}$$

SUPPLEMENTARY NOTE 2

Displacement of a 1-D random walk. For a random walk in one dimension comprised of random displacements R_i, the displacement from the origin after $2k$ steps is

$$D_{2k} = \sum_{i=1}^{2k} R_i$$

If consecutive steps occur in opposite directions, representing a fold, then individual steps can be grouped into k right (positive) and k left (negative) steps:

$$D_{2k} = R_k^+ - R_k^- \quad \text{where} \quad R_k^+ = \sum_{i=1,3,5\ldots}^{2k} R_i, \quad R_k^- = \sum_{i=2,4,6\ldots}^{2k} |R_i|$$

for each r_i drawn from the same distribution. If segment lengths $|R_i|$ are drawn from a gamma distribution with shape parameter $a+1$ and scale parameter θ, consistent with the distribution of facet lengths traversed by a one-dimensional vertical cross-section, then R_k^+ and R_k^- are each distributed according to a gamma distribution with shape parameter $k(a+1)$ and scale parameter θ:

$$|R_i| \sim \Gamma\big(a+1,\theta\big), \quad R_k^+ \sim \Gamma\big(k(a+1),\theta\big), \quad R_k^- \sim \Gamma\big(k(a+1),\theta\big)$$

Thus D_{2k} is the difference of two identically distributed gamma variates. We can obtain the probability density function for D_{2k} through a convolution of the probability density functions of R_k^+ and $-R_k^-$. Let $X = R_k^+$, $Y = R_k^-$, and $Z = D_{2k}$; then

$$\begin{aligned}
f_Z(z) = f_{X-Y}(z) &= \int_{-\infty}^{\infty} f_X(x)f_{(-Y)}(z-x)dx \\
&= \int_{-\infty}^{\infty} f_X(x)f_Y(x-z)dx.
\end{aligned}$$

As $f_X(x)$ and $f_Y(y)$ both have non-negative support,

$$f_Z(z) = \begin{cases} \int_0^\infty f_X(x) f_Y(x-z) dx & \text{for } z \leq 0, \\ \int_0^\infty f_X(y+z) f_Y(y) dy & \text{for } z > 0, \end{cases}$$

where we have chosen the integration variable in the convolution to ensure the arguments of the probability density functions remain positive. With identical gamma distributions

$$f_X(x) = \frac{1}{\theta\Gamma(k(a+1))} \left(\frac{x}{\theta}\right)^{k(a+1)-1} e^{-x/\theta},$$

$$f_Y(y) = \frac{1}{\theta\Gamma(k(a+1))} \left(\frac{y}{\theta}\right)^{k(a+1)-1} e^{-y/\theta},$$

where $\Gamma(k)$ is the gamma function,

$$f_Z(z) = \begin{cases} \frac{e^{z/\theta}}{\theta^2\Gamma(k(a+1))^2} \int_0^\infty \left(\frac{x}{\theta}\right)^{k(a+1)-1} \left(\frac{x-z}{\theta}\right)^{k(a+1)-1} e^{-2x/\theta} dx & \text{for } z \leq 0, \\ \frac{e^{-z/\theta}}{\theta^2\Gamma(k(a+1))^2} \int_0^\infty \left(\frac{y}{\theta}\right)^{k(a+1)-1} \left(\frac{y+z}{\theta}\right)^{k(a+1)-1} e^{-2y/\theta} dy & \text{for } z > 0. \end{cases}$$

The integral above may be solved using the following identity [3]:

$$\int_0^\infty x^{\nu-1}(x+\beta)^{\nu-1} e^{-\mu x} dx = \frac{1}{\sqrt{\pi}} \left(\frac{\beta}{\mu}\right)^{\nu-\frac{1}{2}} e^{\beta\mu/2} \Gamma(\nu) K_{\frac{1}{2}-\nu}\left(\frac{\beta\mu}{2}\right),$$

where $K_\nu(z)$ is the modified Bessel function of the second kind of order ν. This gives

$$f_Z(z) = \frac{1}{\sqrt{\pi}\theta\Gamma(k(a+1))} \left(\frac{|z|}{2\theta}\right)^{k(a+1)-\frac{1}{2}} K_{\frac{1}{2}-k(a+1)}\left(\frac{|z|}{\theta}\right). \quad (4)$$

$f_Z(z)$ should be a valid probability density function, and we can verify it indeed integrates to 1 over its support $z \in [0, \infty)$ using the following identity [4]:

$$\int_0^\infty t^{\alpha-1} K_\nu(t) dt = 2^{\alpha-2} \Gamma\left(\frac{\alpha-\nu}{2}\right) \Gamma\left(\frac{\alpha+\nu}{2}\right).$$

By symmetry about $z = 0$ we can integrate the following:

$$\begin{aligned} &\frac{2}{\sqrt{\pi}\Gamma(k(a+1))} \left(\frac{1}{2}\right)^{k(a+1)-\frac{1}{2}} \int_0^\infty \left(\frac{z}{\theta}\right)^{k(a+1)-\frac{1}{2}} K_{\frac{1}{2}-k(a+1)}\left(\frac{z}{\theta}\right) d\left(\frac{z}{\theta}\right) \\ &\qquad = \frac{2}{\sqrt{\pi}\Gamma(k(a+1))} \left(\frac{1}{2}\right)^{k(a+1)-\frac{1}{2}} 2^{k(a+1)-\frac{3}{2}} \Gamma\left(\frac{2k(a+1)}{2}\right) \Gamma\left(\frac{1}{2}\right) \\ &\qquad = \frac{\Gamma(k(a+1))\Gamma(1/2)}{\sqrt{\pi}\Gamma(k(a+1))} = 1 \end{aligned}$$

as $\Gamma(1/2) = \sqrt{\pi}$. Furthermore, for gamma-distributed steps, the average segment length is given by $(a+1)\theta$. Thus, in our system of a one-dimensional folded strip of total length L_0, k and θ are related as

$$k = \frac{L_0}{2(a+1)\theta}$$

for a strip folded into $2k$ segments. Note that the total length of the walk is distributed as

$$\sum_{i=1}^{2k} |R_i| \sim \Gamma(2k(a+1), \theta)$$

and thus has mean $2k(a+1)\theta = L_0$ and variance $2k(a+1)\theta^2 = L_0\theta$ which tends to zero for small step sizes, improving the approximation of total length.

By using the following identity [5],

$$\int z^{-\nu} K_\nu(z)dz = -2^{-\nu-1}\pi z \csc(\pi\nu)\left[\frac{4^\nu z^{-2\nu}}{(2\nu-1)\Gamma(1-\nu)}{}_1F_2\left(\frac{1}{2}-\nu; 1-\nu; \frac{3}{2}-\nu; \frac{z^2}{4}\right) + \frac{1}{\Gamma(\nu+1)}{}_1F_2\left(\frac{1}{2}; \frac{3}{2}; \nu+1; \frac{z^2}{4}\right)\right] + \text{constant},$$

$f_Z(z)$ may be integrated analytically to obtain an expression for a two-sided survival function as

$$\begin{aligned} S_Z(w; a, \theta) &= P(|Z| > w; w \geq 0) \\ &= 1 - 2\int_0^w f_Z(z)dz \\ &= 1 + \frac{\sqrt{\pi}}{\Gamma(k(a+1))}\left(\frac{w}{\theta}\right)\csc(\pi\nu)\left[\frac{4^\nu}{(2\nu-1)\Gamma(1-\nu)}\left(\frac{w}{\theta}\right)^{-2\nu}{}_1F_2\left(\frac{1}{2}-\nu; 1-\nu, \frac{3}{2}-\nu; \frac{1}{4}\left(\frac{w}{\theta}\right)^2\right)\right. \\ &\left. + \frac{1}{\Gamma(1+\nu)}{}_1F_2\left(\frac{1}{2}; \frac{3}{2}, 1+\nu; \frac{1}{4}\left(\frac{w}{\theta}\right)^2\right)\right], \end{aligned} \tag{5}$$

where $\nu = \frac{1}{2} - k(a+1)$.

SUPPLEMENTARY NOTE 3

Asymptotic Approximation. The main text considers the limit of large k, when the number of steps is large and the step size is small, which permits application of the central limit theorem such that

$$R_k^+, R_k^- \sim \mathcal{N}\Big(k(a+1)\theta, k(a+1)\theta^2\Big),$$

where $\mathcal{N}(\mu, \sigma^2)$ is a normal distribution with mean μ and variance σ^2. Then, the displacement from the origin is

$$D_{2k} = R_k^+ - R_k^- \sim \mathcal{N}(0, 2k(a+1)\theta^2) = \mathcal{N}(0, L_0\theta).$$

Thus, in the limit of large k,

$$f_Z(z;\theta) = \frac{1}{\sqrt{2\pi L_0\theta}}\exp\left(-\frac{z^2}{2L_0\theta}\right), \tag{6}$$

and the corresponding survival function, for $w \geq 0$, is

$$S_Z(w;\theta) = 1 - \text{erf}\left(\frac{w}{\sqrt{2L_0\theta}}\right), \tag{7}$$

where $\text{erf}(z)$ is the error function. This approximation is valid for strongly crumpled experimental samples, but agreement is not guaranteed for samples in the large $\tilde{\Delta}$ regime which have few facets. Thus, we found it important to carry out the exact derivation of Supplementary Equations (4) & (5) to ensure consistency with their respective approximations, Supplementary Equations (6) & (7). Supplementary Fig. 8 shows the increasing agreement of Supplementary Equation (5) and the approximation given by Supplementary Equation (7) for large k.

Next, as explained in the main text, the incremental change in t with crumpling iteration behaves as

$$\delta t \equiv \frac{\partial t}{\partial n} = \alpha\frac{1-\tilde{\Delta}}{\tilde{\Delta}}S_Z(w;\theta), \tag{8}$$

where α is a constant of proportionality, and $\theta = 1/t$. Once again making use of asymptotic approximations, we can integrate $\delta t/S_Z(w;t)$ as follows: In the limit of large t,

$$S_Z(w;t) = 1 - \text{erf}\left(\frac{w}{\sqrt{2L_0/t}}\right) \approx \frac{e^{-w^2 t/2L_0}}{w\sqrt{\pi t/2L_0}}.$$

By a change of variables $u = w\sqrt{t/2L_0}$,

$$I = \int_0^t \frac{dt'}{S_Z(w;t')} = \frac{4L_0\sqrt{\pi}}{w^2}\int_0^u u'^2 e^{u'^2} du',$$

which to leading order in u yields

$$\begin{aligned} I &\approx \frac{2L_0\sqrt{\pi}}{w^2} u e^{u^2} \\ &= \alpha\frac{1-\tilde{\Delta}}{\tilde{\Delta}} n + c \end{aligned}$$

by consequence of Supplementary Equation (8), where c is an integration constant. In order to solve for u, we recall the definition of the Lambert W function, or product logarithm, which gives the inverse solution

$$x = W_0(y)$$

to

$$y = xe^x,$$

where $W_0(y)$ is the principal branch of the Lambert W function valid for real x and y, and positive y. Defining a new variable $z = \left(\alpha\frac{1-\tilde{\Delta}}{\tilde{\Delta}}n + c\right)w^2/L_0\sqrt{2\pi}$, we obtain

$$u = \sqrt{W_0(z^2)/2}$$

or, expressed in terms of t,

$$t = \frac{L_0}{w^2}W_0(z^2).$$

Making a final asymptotic approximation, $W_0(y) \approx \log(y)$, we thus have that

$$\begin{aligned} t &\approx \frac{2L_0}{w^2}\log(z) \\ &= \frac{2L_0}{w^2}\log\left(\frac{w^2}{L_0\sqrt{2\pi}}\left(\alpha\frac{1-\tilde{\Delta}}{\tilde{\Delta}}n + c\right)\right). \end{aligned}$$

With the condition $t(n = 0) = 0$, we obtain the final relation

$$t(n, \tilde{\Delta}; w) = \frac{2L_0}{w^2}\log\left(1 + \frac{\alpha w^2}{L_0\sqrt{2\pi}}\frac{1-\tilde{\Delta}}{\tilde{\Delta}}n\right). \qquad (9)$$

SUPPLEMENTARY NOTE 4

Critical confinement. Our model of a folded one-dimensional strip as a random walk relates geometric incompatibility to the random walk stepping outside a confinement distance w. This critical distance w is dictated by the geometry of the imposed confinement, and the way in which the one-dimensional strip folds into *stacks* of one or more folded *layers* to accommodate its full length within the allowed space. Let m represent the number of spaced stacks, and p the average number of layers per stack, in our strip of length L_0, confined to a rectangular container of width R and height L, $L \leq L_0$. To satisfy the constraint of total length L_0 at any compaction $\tilde{\Delta} = L/L_0$, we must have $mp\sqrt{(L/m)^2 + R^2} = L_0$. The critical width w of facets which would fragment under further confinement is given by $w = L_0/mp = \sqrt{(L/m)^2 + R^2}$. At low confinement, $p \approx 1$, and thus our constraint gives $m = (L_0/R)\sqrt{1-\tilde{\Delta}^2}$, resulting in

$$w(\tilde{\Delta}) = \frac{R}{\sqrt{1-\tilde{\Delta}^2}}. \qquad (10)$$

At high confinement, the collapse of stacks leads to a decrease in m that scales in proportion to L, in turn scaling the number of layers $p \sim 1/L$. Specifically, we can define

$$m(\tilde{\Delta} \to 0) \sim \frac{L}{R},$$
$$p(\tilde{\Delta} \to 0) \sim \frac{L_0}{L}$$

and obtain

$$w(\tilde{\Delta} \to 0) = \frac{L_o}{mp} \sim R$$

which is consistent with our result at low confinement taken to the limit of small L. Thus we use Supplementary Equation (10) throughout. Substituting Supplementary Equation (10) into Supplementary Equation (9), we arrive at an expression for t solely in terms of n and $\tilde{\Delta}$:

$$t(n, \tilde{\Delta}) = \tilde{c}_1(1 - \tilde{\Delta}^2) \log\left(1 + \frac{\tilde{c}_2 n}{\tilde{\Delta}(1 + \tilde{\Delta})}\right), \qquad (11)$$

where $\tilde{c}_1 = 2L_0/R^2$ and $\tilde{c}_2 = \alpha R^2/L_0\sqrt{2\pi}$.

SUPPLEMENTARY REFERENCES

[1] O. Gottesman, J. Andrejevic, C. H. Rycroft, and S. M. Rubinstein, A state variable for crumpled thin sheets, Communications Physics **1**, 70 (2018).
[2] Z. Cheng and S. Redner, Kinetics of fragmentation, Journal of Physics A: Mathematical and General **23**, 1233 (1990).
[3] I. S. Gradshteyn, I. M. Ryzhik, and R. H. Romer, Tables of integrals, series, and products (1988).
[4] Wolfram Research, Inc., Mathematical Functions Site (http://functions.wolfram.com/03.04.21.0116.01), Champaign, IL, 2019.
[5] Wolfram Research, Inc., Mathematical Functions Site (http://functions.wolfram.com/03.04.21.0011.01), Champaign, IL, 2019.
