# Supplementary material for: A model for the fragmentation kinetics of crumpled thin sheets
Source: Nat Commun. 2021 Mar 5;12:1470. doi: 10.1038/s41467-021-21625-2 (PMC7935925; doi:10.1038/s41467-021-21625-2)
Supplement: Supplementary file 2 — Peer Review File [file 41467_2021_21625_MOESM2_ESM.pdf]

Reviewer #1 (Remarks to the Author):

I find the paper interesting. The authors propose a theory for crumpling (in the experiments considered here, crumpling of paper). The theory is based on an analogy to fragmentation, which I believe is original. In essence, this may be viewed as a two-part paper: an earlier manuscript was published with experimental results on crumpling, in which crease length was shown to increase logarithmically with repeated compaction. This follow up paper provides a missing piece: it develops reasonable theoretical arguments to explain the 'slow' logarithmic growth.
The central mechanistic idea is that as facets become smaller, they make the sheet more compliant and therefore lower the rate of subsequent fragmentation.

The analogy to fragmentation is interesting. Besides the key role of defects in fragmentation, as fragments become smaller it becomes more and more energetically difficult to fragment them further: mechanical work becomes increasingly dissipated in internal friction. This is a well-known process in mining and crushing technology.

In light of its originality, I recommend accepting the paper in Nature Comm.

Overall, I find the math in the paper elegant, but perhaps at the expense of a mechanistic understanding. I wish the authors could clarify two/three points.

1) First, although the analogy to fragmentation is quite interesting, I wonder if it is not a bit overplayed in the manuscript.
I understand that the correspondence between crumpling and fragmentation is based on equation (1), and thus on fragmentation kinetics. The maths follow from there. But there are also processes that differentiate crumpling from fragmentation. I would like the authors to highlight in the discussion a few limitations of the analogy. For instance, in dynamic fragmentation what sets the fragment size are Mott's unloading waves. No such process takes place in crumpling.
As a consequence, In dynamic fragmentation the average fragment size is an inverse power law of the loading rate, with a well-defined exponent, robustly observed for a wide range of materials. As far as I understand the equivalent to loading rate in crumpling would the number of compaction iterations. Can the authors comment if the average facet size follows a power law with some universal exponent?

2) Following the above comment, fragmentation analytical models relate fragment size to loading rate and well-defined material properties namely, elastic properties, mass density, and toughness. The authors provide mathematical functions to describe facet areas and length of ridges, but I missed mechanical properties in there. Can the authors comment on where mechanical properties would appear in their theory. I would assume that stiffness and internal friction influence the decrease of average facet area for a given compaction cycle.

3) With increasing compaction cycles, the net number of folds increase. But I wonder if there is some degree of healing, and if that is a limit of the theory. Fig 2b clearly does not show any sign of healing: a couple of well identified facets break up in smaller facets at subsequent iterations. But my interpretation of fig 1 and some data in supplemental is less clear, and left me wondering if for some peculiar cases healing does occur. As an example let us examine fig 1 c, left and right images. The bottom left fragment/facet (left image) seems to become longer(right image) at its base in the x direction. Supplemental material explains that there is a thresholding in ridge curvature to determine ridges and facets, and this may explain whay in some cases facets grow with time. I lacked details in the main text. Does healing indeed occur? Is the threshold for ridge detection constant for all experiments? What is its value? A colour scale in fig 1 b would be useful to interpret results. Are the results and theory sensitive to this threshold?

JF Molinari

Reviewer #2 (Remarks to the Author):

The authors present a model for the evolution of repeatedly crumpled thin sheets. This model is based on the statistics of facet areas and edges and explains a recently obtained experimental result (that the total crease length increases logarithmically with repeated compactions and unfoldings).

This is a very interesting and well-presented manuscript. The theoretical model, based on fragmentation processes, is original and offers a very compelling picture. The paper is of appropriate length and readable by itself, while the Supplementary Information includes the required technical details and derivations. A weakness of the present work is its reliance on the data from Ref. [16] (by some of the same authors). The latter is an experimental exploration of crumpling that found the remarkable logarithmic growth of the crease length that the present work aims to explain. Unfortunately, by not carrying out new experiments, the theory is tested only in a very narrow range. For instance, only one bending rigidity (thickness) is considered, the same experiment is never repeated (so there are no error bars) and even the number of iterations does not always seem to be chosen in the optimal regime, as I will discuss below. On the other hand, the results should appeal to a wide readership and the (though limited in scope) comparison between theory and experiment is satisfactory. Therefore, this work could be appropriate for publication in Nature Communications, provided the specific points raised in the remainder of this report are addressed.

- A key assumption of this work, supported by the results of [16], is that the dynamics does not depend on the crumpling history. However, I believe that what exactly is meant by this independence is not clearly stated in the present manuscript and may rely too much on familiarity with Ref. [16].

- Moreover, on page 4, after eq. (10), it is said that the "added crease length per crumpling iteration [...] is uniquely determined by the sheet's instantaneous state (n,Δ)". This is very confusing, since n does refer to the history. Rather, if I have understood correctly, the state variables should be (ℓ,Δ). This result might not be immediately obvious to a reader from eq. (10), so it could be useful to move equation (18) to right after (10).

- The authors have crumpled the sheets up to 24 times, but only results for 1,2,3 and 24 iterations are shown. Data (surface scans) for the intermediate iterations between 3 and 24 must exist, since they are used in Ref. [16], so I imagine the reason these are not included is because the image analysis (segmentation into facets) has been done by hand and must be very time consuming. Unfortunately, this means that the results are not as convincing as they could have been. The lack of error bars means that it is difficult to judge whether deviations from the theoretical models are significant or fluctuations and data for $n = 24$ is affected by systematic effects (the authors mention that in that case the segmentation is much more difficult to carry out reliably). Indeed, in Figure 4 and, especially, Figure 7 the points for $n = 24$ are far from the curve. This leaves us with just $n=2,3$ to validate the model ($n=1$ also seems to show some deviations in Fig. 4). It would make a big difference if the authors could include data for one or two more values of n.

- Judging from Figure 2, the values of β (or, equivalently, a) seem to change significantly as the crumpling progresses. Is the evolution with n consistent for different compactions? Many values of β are shown in the SI (Figure S6), but some comment on the evolution of this exponent would be warranted in the text. In addition, Figure 3 should report the value of a for the four fits of panel (c).

- Similarily, it is said on page 4 that $\lambda = 1/2$ is consistent for a "wide range" of the data, as shown in Figure 2d. But this figure only shows a single compaction value for $n=1$ and $n=2$. On the other hand, looking at Figure S5, with more complete information, this square-root dependence does not seem to work very well. I think the claim that $\lambda = 1/2$ is consistent should be slightly amended to say that it is a simplification to enable the authors to obtain a more tractable model.

- Related to the above point, maybe I am making a mistake, but comparing Figure 2.d with Figure S5 it seems to me that the two panels of 2d are switched. There is also an inconsistency in notation between both figures (fragmentation from n=j to n=j+1 is labelled by j in one and by j+1 in the other; same goes for S6 and 2e).

- After Eq. (11) it is said that that average number of edges is ne ~ 4.6. But surely this also changes significantly with n and Δ, just like the shape parameter a?.

- An important foundation of the model is relating the total crease length ℓ with the total length of facet edges (the logarithmic growth was experimentally found with the former, the statistical model works with the latter). It is found that the two quantities are proportional, related by (1-Δ). This is justified in an indirect way in the inset to Figure 4, where equation (10) is compared with d. Since equation (10) was already validated in Ref. [16], I think it would be easier just to plot ℓ against d, compared with y = (1-Δ)x, to show the proportionality directly.

- Eq. (13) is only compared with one sample in the paper. It could be interesting to see more examples in the SI.

- Microscopic membranes undergo a spontaneous crumpling transition for very high temperatures [when kT is comparable to the bending energy, see, e.g., Bowick and Travesset, Phys. Rep. 344, 255 (2001) or Nelson, Piran and Weinberg, Statistical Mechanics of Membranes and Surfaces (World Scientific, Singapore, 2004)]. Do the authors believe that a similar model could apply to the kinetics of this thermal crumpling?

Reviewer #3 (Remarks to the Author):

The paper "A model for the fragmentation kinetics of crumpled thin sheets" provides a theoretical model for understanding experimentally observed statistical features of crumpled paper. The model is based on a linear fragmentation model, following the work of Cheng and Redner. The authors use the model to predict facet-size (and crease-length) distributions, and show evidence for fast convergence of different initial conditions to the universal long-time statistics. However, the pinnacle aim of the paper is finding an explanation to the universal logarithmic evolution of total crease length in a repeatedly opened-and-recrumpled paper. For this, the authors must relate an abstract parameter that describes the progression of the fragmentation process to a physical measure, e.g. the number of opening and recrumpling steps. They pursue this goal by introducing a one-dimensional model that maps the current segment size distribution to a random walk process, and checks the probability of this process to leave the confining cell and therefore trigger further fragmentation. Augmented with a few simplifying assumptions, this leads to fragmentation rate whose consistency with experiment is shown.

The model presented here provides an original approach to understanding the crumpling process, that significantly changes the way in which this process is thought of. Furthermore, it demonstrates a highly nontrivial generalization of a theory that was designed and previously used for describing very different systems. This generalization of fragmentation theory could potentially be useful for understanding many other irreversible processes, and thus have an important impact not only in the context of crumpling. The authors have me sold on the idea of using fragmentation theory to describe crumpling. The theoretical modeling in sections II.A-D is quite general, and from a few very reasonable assumptions emerge powerful predictions that are well established by experimental results in Figures 2-5 and in the supplementary data. Also, the paper is very clearly written, and all methods and procedures are explained in detail. However, despite its many strong points, the paper requires major revision prior to publication.

One issue that bothers me is determining the distribution parameter a. Throughout the entire paper, the authors fit this parameter to each and every scan of the sheet separately. However, given the statement of universality, one would expect the value of a to also converge (perhaps after a short preparation-dependent transient) to a predictable value (that may depend on Δ and t). In fact, Figure 5c (if I read it correctly) suggests not only that this is indeed the case, but also that a only depends on t. I would thus expect the authors to have at most one parameter fit per every experimental setup, or even none at all, and not make a fit for every iteration of the same sheet. If indeed a must be fitted to each state separately, I think the authors should tone down some of their claims, and also give an explanation for why a is not a reproducible/predictable parameter.

Alas, the bigger issue in the paper is the validity of the 1D model presented in section II.E. From a theoretical point of view, many of the underlying assumptions of the model are at the very least naive. To point out a few: The very use of a 1D model is suspicious, since the essential geometric problem that initiates crumpling to begin with is 2D. A 1D object, or a 2D object that remains unconfined in one of the spatial dimensions and may therefore remain adopt a "1D configuration", will not crumple. Also, compaction of a previously crumpled state does not result in an independent random walk, since plastic damage drives existing creases to fold in the exact same direction as they were folded before. Also, multi-layer stacking will affect not only S_Z, and thus the total number/length of creases, but also the entire size distribution (and would probably require a nonlinear fragmentation model). Bottom line, from a purely theoretical perspective it is hard to accept the reasoning behind the model as is. I would nonetheless be happy to leap over these gaps and acknowledge that the model works, at least at a phenomenological level, had there been compelling empirical evidence for the success of the model. However, I do not find such strong evidence in the paper. We are only given in this context Figure 7, which is a somewhat indirect measure that may conceal many discrepancies. Moreover, there is a consistent discrepancy that does appear there at small δt, namely at small Δ and large n, which is pretty much the range where I would expect the model to work best (converged to limiting behavior, facet size not yet comparable to thickness).

In order to support the model in section II.E, I think that more compelling evidence is required. First, I would be happy to see some direct experimental validation of specific assumptions in the model. For example, constructing ensembles of 1D sections and check that their distributions match the model's assumptions (not only with regard to crease lengths, but also with regard to crease directions and if possible also position in 3D in the crumpled state). Second, the abstract says "We then demonstrate the capacity of this model to reproduce the characteristic logarithmic scaling of total crease length". Well, why not show exactly that, namely l(n,Δ) from both the model and experiments on the same plot? Such a direct prediction could be extracted from the model, however it differs from the empirical logarithmic relation. The authors may then try to support their claim that in the relevant experimental regime the two models give similar predictions, and outline the limitations of these predictions.

Response to reviewers

We thank the reviewers for carefully reading our manuscript and providing valuable feedback and suggestions. We address individual comments from each reviewer below. In our revised manuscript, new text is highlighted in blue.

Reviewer #1

> *I find the paper interesting. The authors propose a theory for crumpling (in the experiments considered here, crumpling of paper). The theory is based on an analogy to fragmentation, which I believe is original. In essence, this may be viewed as a two-part paper: an earlier manuscript was published with experimental results on crumpling, in which crease length was shown to increase logarithmically with repeated compaction. This follow up paper provides a missing piece: it develops reasonable theoretical arguments to explain the 'slow' logarithmic growth. The central mechanistic idea is that as facets become smaller, they make the sheet more compliant and therefore lower the rate of subsequent fragmentation.*
>
> *The analogy to fragmentation is interesting. Besides the key role of defects in fragmentation, as fragments become smaller it becomes more and more energetically difficult to fragment them further: mechanical work becomes increasingly dissipated in internal friction. This is a well-known process in mining and crushing technology.*
>
> *In light of its originality, I recommend accepting the paper in Nature Comm.*
>
> *Overall, I find the math in the paper elegant, but perhaps at the expense of a mechanistic understanding. I wish the authors could clarify two/three points.*
>
> *1) First, although the analogy to fragmentation is quite interesting, I wonder if it is not a bit overplayed in the manuscript. I understand that the correspondence between crumpling and fragmentation is based on equation (1), and thus on fragmentation kinetics. The maths follow from there. But there are also processes that differentiate crumpling from fragmentation. I would like the authors to highlight in the discussion a few limitations of the analogy. For instance, in dynamic fragmentation what sets the fragment size are Mott's unloading waves. No such process takes place in crumpling.*

We agree with the reviewer that the process of fragmentation does not precisely map to the case of crumpling. In Section II A: Breakup rates, we note one limitation of the model, which assumes a power law relationship in the distribution of the child-to-parent facet areas x/y. Due to a physical lower limit on facet area, we would expect deviation from such scale-invariant behavior as facet size approaches the sheet thickness. One possible way to define this limit is to consider the energetic competition between bending of facets and rigid folding along existing creases, with energy cost of the former proportional to the sheet's bending rigidity, and the latter proportional to crease stiffness [1]. The energy balance of these competing deformations results in a characteristic length scale which varies in proportion to the sheet thickness. We have highlighted this limitation of the current study and make a note of the possible extension in our discussion.

> *As a consequence, In dynamic fragmentation the average fragment size is an inverse power law of the loading rate, with a well-defined exponent, robustly observed for a wide range of*

materials. As far as I understand the equivalent to loading rate in crumpling would the number of compaction iterations. Can the authors comment if the average facet size follows a power law with some universal exponent?

While not a precise power law, we do observe that the average facet size approximately follows a power law relationship with the temporal parameter t, which serves as an effective measure of the maturity of fragmentation. We have revised Fig. 5(c) to more clearly emphasize this relationship.

2) Following the above comment, fragmentation analytical models relate fragment size to loading rate and well-defined material properties namely, elastic properties, mass density, and toughness. The authors provide mathematical functions to describe facet areas and length of ridges, but I missed mechanical properties in there. Can the authors comment on where mechanical properties would appear in their theory. I would assume that stiffness and internal friction influence the decrease of average facet area for a given compaction cycle.

We have done some preliminary studies varying sheet size and thickness to try and understand their effect on the logarithmic accumulation of damage; however, we think this would be the subject of a more detailed follow-up, and focus on presenting a possible theoretical framework for this scaling in the current paper. As noted earlier, a more careful consideration of the lower limit on facet area can also incorporate the sheet's mechanical properties, which determine a length scale that marks the transition between facet bending and folding along existing creases.

3) With increasing compaction cycles, the net number of folds increase. But I wonder if there is some degree of healing, and if that is a limit of the theory. Fig 2b clearly does not show any sign of healing: a couple of well identified facets break up in smaller facets at subsequent iterations. But my interpretation of fig 1 and some data in supplemental is less clear, and left me wondering if for some peculiar cases healing does occur. As an example let us examine fig 1 c, left and right images. The bottom left fragment/facet (left image) seems to become longer(right image) at its base in the x direction. Supplemental material explains that there is a thresholding in ridge curvature to determine ridges and facets, and this may explain whay in some cases facets grow with time. I lacked details in the main text. Does healing indeed occur? Is the threshold for ridge detection constant for all experiments? What is its value? A colour scale in fig 1 b would be useful to interpret results. Are the results and theory sensitive to this threshold?

The reviewer raises an important concern; however, we first point out that the left and right images of Fig. 1(c) refer to two distinct sheets crumpled at different compaction ratios and are unrelated in their crease patterns; we have updated the figure and caption to clarify this. Thresholding for ridge detection is relevant for automated segmentation, though the results presented in the main text are all manually labeled. However, in light of the reviewer's question, we have performed a simple analysis and affirm that healing is indeed very minimal, and note these results in the Supplementary Information, Section II.A. We can make a quantitative prediction about the extent of healing by overlaying two crease patterns from successive crumples $n-1$ and n of the same sheet, and measuring the length of creases present in $n-1$ which do not appear in n. After doing this, we see that the percent of "healed" creases is less than 5% of any crease pattern, and in fact the fraction is typically within 1% for moderately to highly dense patterns. Thus while some creases can soften over repeated crumples, and the result of unfolding, scanning, and calculating the curvature could contribute to the appearance of healing, we are confident this is a small effect which does not greatly impact the dominant trends in the data.

Reviewer #2

The authors present a model for the evolution of repeatedly crumpled thin sheets. This model is based on the statistics of facet areas and edges and explains a recently obtained experimental result (that the total crease length increases logarithmically with repeated compactions and unfoldings).

This is a very interesting and well-presented manuscript. The theoretical model, based on fragmentation processes, is original and offers a very compelling picture. The paper is of appropriate length and readable by itself, while the Supplementary Information includes the required technical details and derivations. A weakness of the present work is its reliance on the data from Ref. [16] (by some of the same authors). The latter is an experimental exploration of crumpling that found the remarkable logarithmic growth of the crease length that the present work aims to explain. Unfortunately, by not carrying out new experiments, the theory is tested only in a very narrow range. For instance, only one bending rigidity (thickness) is considered, the same experiment is never repeated (so there are no error bars) and even the number of iterations does not always seem to be chosen in the optimal regime, as I will discuss below. On the other hand, the results should appeal to a wide readership and the (though limited in scope) comparison between theory and experiment is satisfactory. Therefore, this work could be appropriate for publication in Nature Communications, provided the specific points raised in the remainder of this report are addressed.

- A key assumption of this work, supported by the results of [16], is that the dynamics does not depend on the crumpling history. However, I believe that what exactly is meant by this independence is not clearly stated in the present manuscript and may rely too much on familiarity with Ref. [16].

We thank the reviewer for pointing this out. We have added a clarifying statement in the introductory paragraph to clarify this: "Notably, the incremental damage added upon re-crumpling the sheet was found to be independent of the sheets' crumpling history—the sequence of prior compactions performed to produce the current crease network. Rather, the added crease length is determined only by the current total crease length and the new compaction depth."

- Moreover, on page 4, after eq. (10), it is said that the "added crease length per crumpling iteration [...] is uniquely determined by the sheet's instantaneous state $(n, \tilde{\Delta})$". This is very confusing, since n does refer to the history. Rather, if I have understood correctly, the state variables should be $(\ell, \tilde{\Delta})$. This result might not be immediately obvious to a reader from eq. (10), so it could be useful to move equation (18) to right after (10).

This is a helpful suggestion, and we have clarified the state variables as $(\ell, \tilde{\Delta})$ and made the suggested reordering in the equations. In the revised manuscript, Eq. (11) and (12) now give the evolution of ℓ and the incremental change $\delta\ell$, respectively, for the empirical model of Ref. [16].

- The authors have crumpled the sheets up to 24 times, but only results for 1, 2, 3 and 24 iterations are shown. Data (surface scans) for the intermediate iterations between 3 and 24 must exist, since they are used in Ref. [16], so I imagine the reason these are not included is because the image analysis (segmentation into facets) has been done by hand and must be very time consuming. Unfortunately, this means that the results are not as convincing as they could

have been. The lack of error bars means that it is difficult to judge whether deviations from the theoretical models are significant or fluctuations and data for $n = 24$ is affected by systematic effects (the authors mention that in that case the segmentation is much more difficult to carry out reliably). Indeed, in Figure 4 and, especially, Figure 7 the points for $n = 24$ are far from the curve. This leaves us with just $n = 2, 3$ to validate the model ($n = 1$ also seems to show some deviations in Fig. 4). It would make a big difference if the authors could include data for one or two more values of n.

We have performed segmentations of additional crumpling iterations using our automated approach, and now include these extended results in the Supplementary Information. In our comparison of the empirical and derived models for $\delta\ell$ and ℓ in Fig. 7, we have also included the auto-segmented data as a reference. Though we acknowledge that crease networks with fewer creases are susceptible to greater discrepancies between manual and automated segmentation, denser and more developed crease networks are generally closer in agreement both between the two segmentation approaches, and in their consistency with the logarithmic scaling, as shown in Figure 7.

- Judging from Figure 2, the values of β (or, equivalently, a) seem to change significantly as the crumpling progresses. Is the evolution with n consistent for different compactions? Many values of β are shown in the SI (Figure S6), but some comment on the evolution of this exponent would be warranted in the text. In addition, Figure 3 should report the value of a for the four fits of panel (c).

Based on the suggestions of Reviewer #3, we have revised the way the parameter a is determined: Rather than fitting a value of a independently for each sample, we identify a universal relationship between a and t with a single fitting parameter that then allows a to be calculated for all distributions, given by Eq. (8). A discussion of this is now provided in Section II C: Ridge length statistics, and a plot of a vs. t is included in Fig. 4(a). Indeed this approach much better clarifies the evolution of this exponent, which grows slowly as $t^{1/2}$. We have also added the corresponding value of a for each distribution in Fig. 3(c).

- Similarily, it is said on page 4 that $\lambda = 1/2$ is consistent for a "wide range" of the data, as shown in Figure 2d. But this figure only shows a single compaction value for $n = 1$ and $n = 2$. On the other hand, looking at Figure S5, with more complete information, this square-root dependence does not seem to work very well. I think the claim that $\lambda = 1/2$ is consistent should be slightly amended to say that it is a simplification to enable the authors to obtain a more tractable model.

We agree with the reviewer that the choice of $\lambda = 1/2$ is advantageous for obtaining a more tractable model; we now explicitly acknowledge this in Section II B, and have elaborated further on our justification for this choice in Section II A.

- Related to the above point, maybe I am making a mistake, but comparing Figure 2.d with Figure S5 it seems to me that the two panels of 2d are switched. There is also an inconsistency in notation between both figures (fragmentation from $n = j$ to $n = j + 1$ is labelled by j in one and by $j + 1$ in the other; same goes for S6 and 2e).

We thank the reviewer for pointing this out; this was indeed a mistake and the panels in Fig. 2 are now correctly ordered.

- After Eq. (11) it is said that that average number of edges is $n_e \sim 4.6$. But surely this also changes significantly with n and $\tilde{\Delta}$, just like the shape parameter a?.

We have added clarification for this as a panel in Fig. 4(b), which demonstrates that 4.2 is a valid estimate for the average number of edges across all samples. (Note a slight adjustment to this value occurred in our revision and new approach for determining the shape parameter a.) Equation (14) of the revised manuscript gives a prediction for the relationship between the total crease length as a function of $(1-\tilde{\Delta})t/(a+1)$, such that plotting a measured ℓ from each sample vs. $(1-\tilde{\Delta})t/(a+1)$ gives a line whose slope is $n_e/2$, half the average number of edges per facet. We find that the slope of the line is ≈ 2.1, which gives $n_e = 4.2$. Though this is not a direct way of calculating the number of edges per facet, we believe it is a suitable estimate, relying on the moments of distributions that show strong agreement with the data.

- An important foundation of the model is relating the total crease length ℓ with the total length of facet edges (the logarithmic growth was experimentally found with the former, the statistical model works with the latter). It is found that the two quantities are proportional, related by $(1-\tilde{\Delta})$. This is justified in an indirect way in the inset to Figure 4, where equation (10) is compared with d. Since equation (10) was already validated in Ref. [16], I think it would be easier just to plot ℓ against d, compared with $y = (1-\tilde{\Delta})x$, to show the proportionality directly.

We thank the reviewer for this suggestion; in Fig. 4(c), we now provide a plot of the new $\ell(t,\tilde{\Delta})$ from Eq. (14) which is a function of t, plotted against the empirical relation $\ell(n,\tilde{\Delta})$ from Eq. (11), which is a function of n, to show their proportionality directly.

- Eq. (13) is only compared with one sample in the paper. It could be interesting to see more examples in the SI.

We now provide the distribution of segment lengths for all manually segmented examples in Fig. S5 in the Supplementary Information.

- Microscopic membranes undergo a spontaneous crumpling transition for very high temperatures [when kT is comparable to the bending energy, see, e.g., Bowick and Travesset, Phys. Rep. 344, 255 (2001) or Nelson, Piran and Weinberg, Statistical Mechanics of Membranes and Surfaces (World Scientific, Singapore, 2004)]. Do the authors believe that a similar model could apply to the kinetics of this thermal crumpling?

We thank the reviewer for bringing these studies of thermally crumpled membranes to our attention; while we do not have extensive familiarity with this related topic, to our best knowledge the crumpling transition is characteristic of phantom membranes, and is suppressed for self-avoiding membranes in the case of purely thermally-driven dynamics. We would need to think more about the direct analogy between thermal membranes and macroscopic crumpled sheets, but it is possible that the correlation length of the surface normal in thermally crumpled membranes could be related to the mean facet area in our model, and thereby offer a route for applying a similar fragmentation analysis to this system.

Reviewer #3

The paper "A model for the fragmentation kinetics of crumpled thin sheets" provides a theoretical model for understanding experimentally observed statistical features of crumpled paper. The model is based on a linear fragmentation model, following the work of Cheng and Redner. The authors use the model to predict facet-size (and crease-length) distributions, and show evidence for fast convergence of different initial conditions to the universal long-time statistics. However, the pinnacle aim of the paper is finding an explanation to the universal logarithmic evolution of total crease length in a repeatedly opened-and-recrumpled paper. For this, the authors must relate an abstract parameter that describes the progression of the fragmentation process to a physical measure, e.g. the number of opening and recrumpling steps. They pursue this goal by introducing a one-dimensional model that maps the current segment size distribution to a random walk process, and checks the probability of this process to leave the confining cell and therefore trigger further fragmentation. Augmented with a few simplifying assumptions, this leads to fragmentation rate whose consistency with experiment is shown.

The model presented here provides an original approach to understanding the crumpling process, that significantly changes the way in which this process is thought of. Furthermore, it demonstrates a highly nontrivial generalization of a theory that was designed and previously used for describing very different systems. This generalization of fragmentation theory could potentially be useful for understanding many other irreversible processes, and thus have an important impact not only in the context of crumpling. The authors have me sold on the idea of using fragmentation theory to describe crumpling. The theoretical modeling in sections II.A-D is quite general, and from a few very reasonable assumptions emerge powerful predictions that are well established by experimental results in Figures 2-5 and in the supplementary data. Also, the paper is very clearly written, and all methods and procedures are explained in detail. However, despite its many strong points, the paper requires major revision prior to publication.

One issue that bothers me is determining the distribution parameter a. Throughout the entire paper, the authors fit this parameter to each and every scan of the sheet separately. However, given the statement of universality, one would expect the value of a to also converge (perhaps after a short preparation-dependent transient) to a predictable value (that may depend on $\tilde{\Delta}$ and t). In fact, Figure 5c (if I read it correctly) suggests not only that this is indeed the case, but also that a only depends on t. I would thus expect the authors to have at most one parameter fit per every experimental setup, or even none at all, and not make a fit for every iteration of the same sheet. If indeed a must be fitted to each state separately, I think the authors should tone down some of their claims, and also give an explanation for why a is not a reproducible/predictable parameter.

We thank the reviewer for this suggestion, and we hope our revisions better clarify the evolution of a. First, we have revised the way the parameter a is determined: Rather than fitting a value of a independently for each sample, we identify a universal relationship between a and t with a single fitting parameter that then allows a to be calculated for all distributions, given by Eq. (8). A discussion of this is now provided in Section II C: Ridge length statistics, and a plot of a vs. t is included in Fig. 4(a). We do observe that a evolves predictably as $t^{1/2}$, consistent with our expectation that a changes less and less as the crease network becomes more mature.

Alas, the bigger issue in the paper is the validity of the 1D model presented in section II.E. From a theoretical point of view, many of the underlying assumptions of the model are at the very least naive. To point out a few: The very use of a 1D model is suspicious, since the essential geometric problem that initiates crumpling to begin with is 2D. A 1D object, or a 2D object that remains unconfined in one of the spatial dimensions and may therefore remain adopt a "1D configuration", will not crumple. Also, compaction of a previously crumpled state does not result in an independent random walk, since plastic damage drives existing creases to fold in the exact same direction as they were folded before. Also, multi-layer stacking will affect not only S_Z, and thus the total number/length of creases, but also the entire size distribution (and would probably require a nonlinear fragmentation model). Bottom line, from a purely theoretical perspective it is hard to accept the reasoning behind the model as is. I would nonetheless be happy to leap over these gaps and acknowledge that the model works, at least at a phenomenological level, had there been compelling empirical evidence for the success of the model. However, I do not find such strong evidence in the paper. We are only given in this context Figure 7, which is a somewhat indirect measure that may conceal many discrepancies. Moreover, there is a consistent discrepancy that does appear there at small δt, namely at small $\tilde{\Delta}$ and large n, which is pretty much the range where I would expect the model to work best (converged to limiting behavior, facet size not yet comparable to thickness).

In order to support the model in section II.E, I think that more compelling evidence is required. First, I would be happy to see some direct experimental validation of specific assumptions in the model. For example, constructing ensembles of 1D sections and check that their distributions match the model's assumptions (not only with regard to crease lengths, but also with regard to crease directions and if possible also position in 3D in the crumpled state).

We thank the reviewer for the feedback, and we fully understand the reviewer's concerns about the assumptions that we have employed. However, our work represents an honest attempt to gain theoretical insight into a complex problem, and we needed to make these assumptions to obtain something analytically tractable. While it is likely that the model could be subsequently refined, we think that its current form provides new insight into the physics of crumpling: The fragmentation model explains the universality seen in crease patterns, and the random walk model provides an explanation for why the growth of creases scales approximately logarithmically.

The reviewer raises concerns about the link between the 2D data and the 1D model, but this is already quantitatively discussed in our paper, and we think that our calculations are sound. We begin by calculating distributions of facet areas in 2D, and at the start of Sec. II.E we derive the associated distribution of 1D segment lengths on vertical transects in Eq. (15). This calculation correctly accounts for the fact that facets with larger area will more likely be observed in a transect. We already included an example in Fig. 6(b) that demonstrates that the distributions of facet segment lengths indeed match[1] our calculated 1D distribution with no additional fitting parameters.

Since the sheets are wrapped around the inside of the piston, we think that viewing each sheet as a collection of 1D vertical cross-sections is a reasonable perspective. We use the 1D cross-sections as a way to estimate the rate of subsequent fragmentation. In our model, it would indeed be the case that nearby 1D cross-sections are not independent, since a large facet may be responsible for

[1] A slight discrepancy can be seen in Fig. 6(b) for values of r less than 10^{-3}, but this is expected and emerges due to the limit on scanning resolution. The bulk of the distribution matches accurately. Similar features are visible in some of the raw facet area data, such as Fig. S4.

several long segment lengths in nearby cross-sections. However, our calculation of the rate does not require that the 1D random walks are independently sampled; all that is required is an averaged value over all 1D cross-sections.

Based on feedback from Reviewer #2, we now include substantially more data in Figure 7, which features both manually segmented data and automatically segmented data. This new data increases the strength of agreement. The reviewer is correct that there are some small discrepancies for small δt, but this is a difficult limit to probe, since we approach the limit where facets become so small that they are not properly resolved in our scans.

> *Second, the abstract says "We then demonstrate the capacity of this model to reproduce the characteristic logarithmic scaling of total crease length". Well, why not show exactly that, namely $l(n, \tilde{\Delta})$ from both the model and experiments on the same plot? Such a direct prediction could be extracted from the model, however it differs from the empirical logarithmic relation. The authors may then try to support their claim that in the relevant experimental regime the two models give similar predictions, and outline the limitations of these predictions.*

Following the reviewer's suggestion, we have extended our manuscript to include a derivation of $t(n, \tilde{\Delta})$ which follows from integrating the model for incremental change in t presented in Eq. (18). This derivation is provided in detail in the SI Section IV, and the result is provided in the main text in Eq. (21). Taken together, Eq. (21) and the relation for $\ell(t, \tilde{\Delta})$ given by Eq. (14) provide a new model for $\ell(n, \tilde{\Delta})$ which may be compared to the empirical relation for $\ell(n, \tilde{\Delta})$ presented in Ref. [16] (restated in Eq. (11)). In our revised Fig. 7, we now show the agreement between $\delta\ell$ and ℓ and their respective measured values from experiment, for both the empirical model (Ref. [16]) and the new model derived in this work. We hope this more clearly illustrates that the two models perform comparably, and affirms the suitability of the logarithmic scaling.

References

[1] F. Lechenault, B. Thiria, M. Adda-Bedia, Mechanical response of a creased sheet, Physical Review Letters 112 (24) (2014) 244301.

Reviewer #1 (Remarks to the Author):

I am fine with the response provided by the authors.
I recommend accepting the article.

Reviewer #2 (Remarks to the Author):

In my original report, I concluded that this was an interesting paper but raised a number of points about the statistical analysis (in particular, the computation of parameters such as a) and the interpretation of the results. I find that in this revised version the authors have done a lot of work to make their analysis more solid and their presentation clearer and more complete. I welcome in particular the changes to the figures and the additions to the supplemental information.

As pointed out by other reviewers and by myself, the model discussed in this paper (and its successive simplifications) are not fully justified theoretically, but I believe it provides compelling new insight into the physics of crumpling and will serve as the foundation for more refined studies. In light of this and of the originality of the study, I recommend publication of the work in this revised form.

A final side note: in my original review, I asked the authors whether they had some comments on the possible relation or relevance of this study to the physics of thermal crumpling. The correctly pointed out that this phase transition has only been demonstrated for phantom membranes. However, I would like to add that self-avoiding membranes, even if they do not experience fully developed crumpling (which is in question) will at least approach the crumpled phase. This is especially true for membranes with perforations, where self avoidance could be less of an obstacle. In this context, it has recently been observed [D. Yllanes, D.R. Nelson, M.J. Bowick, Phys. Rev. E 100, 042112 (2019)] that in a simple "frame" geometry the membrane approaches the crumpled phase through a sequence of origami-like folds at decreasing length scales. This seemed to me to be qualitatively reminiscent of the process discussed in this work.

Reviewer #3 (Remarks to the Author):

I am happy with the revised version of the paper and recommend its publication in Nature Communications.

In particular, the additional data in Fig. 7 and the universal scaling of the a parameter significantly strengthen the results. Admittedly, I still find several theoretical assumptions in the 1D model hard to justify. However, the authors did a good job in demonstrating that phenomenologically the model works in producing accurate quantitative predictions to the measures that are at the focus of this paper. Whether this is because the 1D model is indeed sound or because these predictions are insensitive to its details -- does not matter. At the end of the day, these are strong nontrivial predictions for key observations in the statistics of crumpled paper, and the fragmentation model nails it.

Response to reviewers

We thank the reviewers for their additional comments about our paper. Reviewers 1 and 3 request no further changes. We have made a small modication to address a comment from Reviewer 2:

> *A final side note: in my original review, I asked the authors whether they had some comments on the possible relation or relevance of this study to the physics of thermal crumpling. The correctly pointed out that this phase transition has only been demonstrated for phantom membranes. However, I would like to add that self-avoiding membranes, even if they do not experience fully developed crumpling (which is in question) will at least approach the crumpled phase. This is especially true for membranes with perforations, where self avoidance could be less of an obstacle. In this context, it has recently been observed [D. Yllanes, D.R. Nelson, M.J. Bowick, Phys. Rev. E 100, 042112 (2019)] that in a simple "frame" geometry the membrane approaches the crumpled phase through a sequence of origami-like folds at decreasing length scales. This seemed to me to be qualitatively reminiscent of the process discussed in this work.*

We now describe the work by Yllanes *et al.* in the discussion section.
